# Time From Authorization by the US Food and Drug Administration to Medicare Coverage for Novel Technologies

**DOI:** 10.1001/jamahealthforum.2023.2260

**Published:** 2023-08-04

**Authors:** Zachary A. Sexton, Juliana R. Perl, Henry R. Saul, Artem A. Trotsyuk, Jan B. Pietzsch, Sandra Waugh Ruggles, Margaret C. Nikolov, Kevin A. Schulman, Josh Makower

**Affiliations:** 1 Stanford Byers Center for Biodesign, Stanford University, Stanford, California; 2 Center for Biomedical Ethics, Stanford University, Stanford, California; 3 Wing Tech Inc, Menlo Park, California; 4 Summit Rock Strategy Consulting, Sunnyvale, California; 5 Clinical Excellence Research Center, Department of Medicine, Stanford University, Stanford, California; 6 Graduate School of Business, Stanford University, Stanford, California; 7 Department of Bioengineering, Stanford University, Stanford, California; 8 Department of Cardiovascular Medicine, Stanford University, Stanford, California

## Abstract

**Importance:**

A wide variety of novel medical diagnostics and devices are determined safe and effective by the US Food and Drug Administration (FDA) each year, but to our knowledge the literature lacks evidence documenting how long it takes to establish new Medicare coverage for these technologies.

**Objective:**

To measure time from FDA authorization to at least nominal Medicare coverage for technologies requiring a new reimbursement pathway.

**Design, Setting, and Participants:**

In this cross-sectional study, public databases were used to associate each technology to billing codes, determine the effective date of each code and Medicare coverage decisions, and stratify by the maturity of the Medicare coverage. At least nominal coverage was defined as achievement of explicit coverage milestones through a national coverage determination, local coverage determinations by Medicare administrative contractors, or by implicit coverage aligned to a new billing code. Characterization by product type (acute treatment, chronic or ongoing treatment, diagnostic assay, and diagnostic device), manufacturer size, and evidence level were assessed for association with coverage achievement. The study included new product applications authorized by the FDA through the premarket approval pathway, the de novo pathway, or with breakthrough designation in the 510(k) pathway from January 1, 2016, to December 31, 2019. Data analysis took place between May 1, 2022, and December 31, 2022.

**Main Outcome Measurement:**

Time from FDA authorization to the first coverage milestone.

**Results:**

Among 281 identified technologies in the total sample, 64 (23%) were deemed novel technologies based on the absence of coverage determinations and/or the use of temporary or miscellaneous billing codes. Twenty-eight of 64 technologies (44%) successfully achieved explicit or implicit coverage following FDA authorization. The median time to at least nominal coverage for the analysis cohort was 5.7 years (90% CI, 4.4-NA years). Analysis of time-to-coverage data highlighted company size (log-rank *P*<.001) and product type (log-rank *P* = .01) as significant covariates associated with coverage achievement. No association was observed for technologies with level 1 evidence at FDA authorization and subsequent coverage milestone achievement (log-rank *P* = .40).

**Conclusions and Relevance:**

In this cross-sectional study of 64 novel technologies, only 28 (44%) achieved coverage milestones over the study timeline. The several-year period observed to establish at least nominal coverage suggests existing coverage processes may affect timely reimbursement of new technologies.

## Key Points

**Question:**

How long does it take to establish Medicare coverage for novel medical technologies?

**Findings:**

In this cross-sectional study, 64 devices and diagnostics authorized by the US Food and Drug Administration through premarket approval and de novo pathways between 2016 and 2019 required establishment of new Medicare coverage; at least nominal explicit or implicit Medicare coverage supportive of patient access was achieved by 28 (44%) within a median of 5.7 years.

**Meaning:**

Lengthy processes to establish Medicare coverage warrant attention; timelines for coverage achievement can be used to inform new policy strategies.

## Teaser

This cross-sectional study examines contemporary evidence about the progress to Medicare coverage for a cohort of novel US Food and Drug Administration-authorized technologies for which a reimbursement pathway has not already been established.

## Introduction

In 2022, more than 3000 medical devices and diagnostics were introduced into the US health care system through the US Food and Drug Administration’s (FDA) Center for Devices and Radiological Health (CDRH).^1^ Analysis of medical device development has often focused on timelines to FDA authorization.^2^ Although achieving FDA authorization provides a manufacturer legal authorization for market access, uptake of these technologies and therefore meaningful patient access, is dependent on achieving reimbursement by health insurers.

The reimbursement process is how the insurer makes a determination of whether and how it will pay for a health care service. This determination involves 3 steps: coverage, coding, and payment. Coverage asks the question of whether the novel technology meets criteria established in the insurance contract as a covered benefit for patients. By statute, the coverage standard for the Medicare program is that services must be “reasonable and necessary” and fall within a benefit category defined by law. Coding is a process of creating a unique identifier in the claims system for the technology. Generally, coding of medical procedures is developed by the American Medical Association (AMA), whereas coding for medical devices is developed by the Centers for Medicare & Medicaid Services (CMS). Each year, more than 200 new codes are created to accurately reflect changes in the health care landscape.^3^ The creation of these new codes requires advocacy by physician societies and a review and publication process that lasts between 12 and 15 months.^4,5^ Payment assigns a monetary amount for the provision of covered (and coded) medical items and services.

The most influential coverage decisions are often made by CMS because they often precede private health plans.^6^ These CMS coverage determinations may be explicit, implicit, or made through claim-by-claim adjudication. National coverage determinations (NCDs) are explicit coverage decisions made at the national level by Medicare. These determinations are infrequent, with only 3 to 4 NCDs initiated annually in 2018 to 2021.^7^ Medicare administrative contractors (MACs) make explicit coverage determinations at the regional level through local coverage determinations (LCDs). Both NCDs and LCDs must be followed by traditional and Medicare Advantage organizations. Manufacturers typically initiate these coverage determinations through requests to CMS which include assessments of clinical evidence and indications for use. .

Medical technologies lacking an NCD or LCD can still have implicit coverage if the technology aligns with an established code that describes its use in clinical practice. However, reimbursement of novel medical technologies is unreliable when no coverage determinations exist, and when implicit coverage cannot be linked to an appropriate code. In these situations, temporary common procedural terminology (CPT) codes, unlisted codes, or miscellaneous codes are used to submit claims, and case-by-case adjudication may be necessary. Using these codes introduces hurdles to reimbursement and may require physicians to navigate lengthy administration processes to receive payment. Meanwhile, patients are more likely to incur out-of-pocket costs.^8,9,10,11^

Beyond Medicare, different reimbursement paradigms exist for Medicaid or private insurers owing to the nature of state-based and employer-sponsored insurance. Coverage determinations through employer-sponsored private insurance plans vary widely; only roughly half of private determinations align with NCDs, a quarter are more restrictive, and a quarter are less restrictive.^12^ In addition, Medicaid and the Children’s Health Insurance Program (CHIP) cover more than 40% of births, more than 41 million children, and more than 50 million adult beneficiaries, yet generally lack explicit policies for considering new devices and diagnostics.^13,14^ Thus, policy initiatives focused on Medicare also affect these programs and populations.

Although a recent survey of investors and manufactures found that time to national Medicare coverage following FDA authorization is on average 4.7 years, to our knowledge, there is little to no literature objectively quantifying timelines of the reimbursement process.^15^ The objective of the current study was to provide contemporary evidence about the progress to Medicare coverage for a cohort of new FDA-authorized technologies for which a reimbursement pathway has not already been established.

## Methods

In this cross-sectional study, a convergent parallel design methodology was used consisting of objective analyses for all technologies meeting specific FDA regulatory pathways and authorization year criteria, and qualitative interviews with a convenience sample of market access experts at 25 manufacturers (eMethods in Supplement 1). This design provided insight into coverage, coding, and payment achievement while retaining an inclusive analysis set. Based on the information provided, the Stanford University School of Medicine institutional review board determined that this research did not involve human participants as defined in 45 CFR 46.102(f) or 21 CFR 50.3(g), and therefore did not require written informed consent. The Strengthening the Reporting of Observational Studies in Epidemiology (STROBE) reporting guidelines were used to ensure the reporting of this study (Supplement 1).^16^

The FDA database was screened for original applications that received market authorization during the enrollment period between January 1, 2016, and December 31, 2019. The cohort included technologies approved or cleared through the FDA’s premarket approval (PMA) and de novo pathways, as well as 510(k) devices with breakthrough designation, resulting in 153, 124, and 4 products, respectively, for a total initial cohort of 281 technologies. These 3 FDA pathways for authorization were considered likely to contain the most novel technologies based on the lack of comparable device predicates. The follow-up period extended from January 1, 2016, to December 31, 2022, such that all technologies in the analysis set were at least 3 years from FDA authorization. Three milestones were determined as the transition from claim-by-claim adjudication to at least nominal coverage: a new NCD, positive LCDs from a plurality (3/7) of MACs, or implicit coverage through 1 or more new Healthcare Common Procedure Coding System (HCPCS) level 1 or level 2 codes specific to the technology (Box).

Box. **Definition of Milestones That Establish at Least Nominal Medicare Coverage^a^**

1. Explicit coverage through
  National Coverage Determination (NCD)
  Local Coverage Determinations (LCDs) via
    ≥3 of 7 MACs
    ≥2 of 4 durable medical equipment (DME) MACs
    MolDx
2. Implicit coverage when billed with
  HCPCS Level 1 codes (CPT I Codes)
  HCPCS Level 2 codes (excluding C-, K-codes)

Abbreviations: CPT, common procedural terminology; HCPCS, Healthcare Common Procedure Coding System; MACs, Medicare administrative contractors; MolDx, molecular diagnostic services.

^a^
Thresholds of at least nominal Medicare coverage include multiple coverage milestones defined from data collected during interviews with industry experts and from the authors expertise with coding, coverage, and payment of novel technologies (eMethods in Supplement 1).

For all technologies in the analysis cohort, associated billing codes were established via online billing and coding forums, clinical labratory submission instructions, and manufacturer-provided reimbursement information. Codes were reviewed for accuracy by 2 study authors (Z.A.S. and S.W.R.). Technologies were excluded if they had no associated code, were billed with tracking codes (eg, *International Statistical Classification of Diseases and Related Health Problems, Tenth Revision [ICD-10]*-PCS or C-code), had an indication for use restricted to inpatient care, or were capital equipment, software, or consumer-facing products otherwise lacking a Medicare benefit category. Using the Medicare Coverage Database, a designated coding database (Find-A-Code), and FDA documentation on authorized indications, the identified CPT and HCPCS codes were used to locate all CMS coverage literature through Medicare LCDs, billing and coding articles, and NCDs.^17,18,19,20,21,22^ Revision history information was reviewed to determine accurate time point association of coding and coverage. Additional technologies were excluded from analysis when at least nominal coverage was effective before FDA authorization.

Clinical evidence included in the FDA authorization materials was reviewed independently by 2 study authors (Z.A.S. and J.R.P.) on clinicaltrials.gov to ensure consensus in assessing level 1 evidence. Authors referred to the Oxford Center for Evidence-Based Medicine evidence standards for level 1 evidence definitions concerning interventional and diagnostic technologies.^23^ Level 1 evidence for diagnostics is generally defined by validation (as opposed to exploratory) studies with clear reference standards, whereas for devices it is defined by randomized clinical trials evaluating technology performance over the existing clinical standard or a sham control. Technology types were defined as 4 broad categories including diagnostic assays, diagnostic devices, acute treatments, and chronic or ongoing treatments, which were determined from indications for use statements in FDA authorizations. To facilitate subanalyses by manufacturer size, company size was researched from current public data sources and classified into small (<200 employees) and large manufacturers.

### Statistical Analysis

Coverage probabilities at 1, 3, and 5 years were estimated using the standard 1-sample method for population proportions. Time to coverage was computed as the difference between date of FDA authorization and date at which an at least nominal coverage milestone was reached; if no such milestone was achieved by the close of the study period (December 31, 2022), the data were considered lost to follow-up at that point (right censored at that time). Kaplan-Meier curves were estimated to demonstrate achievement of coverage over time. The log-rank test was used to assess factors associated with time to coverage, including strength of clinical evidence, type of technology, and size of commercial manufacturer. All tests were conducted using a standard α = .10 type I error rate. All statistical analyses were performed using R statistical software with survival package (version 4.2.3, R Project for Statistical Computing).

## Results

**Figure 1.**
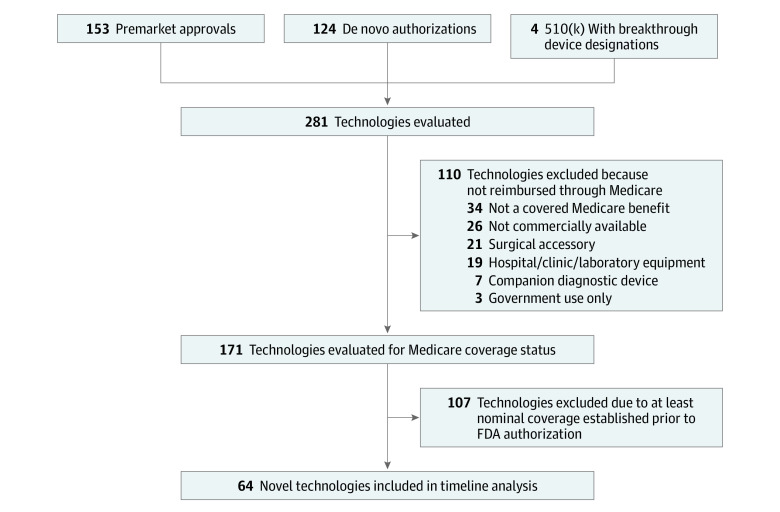
Flowchart of Medicare Coverage for Technologies in the Analysis Set

The total study cohort included 281 technologies (Figure 1). Products spanned 20 FDA advisory committees with cardiovascular (n = 66, 23%) and microbiology (n = 33, 12%) accounting for the largest number of product authorizations (eTable 1 in Supplement 1); products were distributed among 4 categories: diagnostic assays (n = 75, 27%), diagnostic devices (n = 47, 17%), acute treatment devices (n = 86, 30%), and chronic or ongoing treatment devices (n = 73, 26%) (eTable 2 in Supplement 1).

One hundred ten technologies (39%) were not directly reimbursed by Medicare. These included surgical supplies and supplies for in-patient hospitalizations such as intubation kits and surgical sealants, capital equipment or software associated with broad patient use cases, companion diagnostics for cancer therapeutics, and consumer-oriented technologies that are noncovered benefits such as hearing aids. Among these technologies were also those that did not fall under an established Medicare benefit category. One hundred seventy-one technologies (61%) were evaluated for coverage status. One hundred seven technologies (38%) used coding and coverage pathways that were established prior to the FDA authorization. These technologies often represent improvements to technologies that have established clinical value such as diagnostic assays for viral infections and stents. The remaining 64 technologies (23%) did not have at least nominal coverage and were considered novel. These novel technologies were further analyzed for timing to coverage milestones. Manufacturer reimbursement information listing temporary CPT category 3 codes, miscellaneous, or unlisted codes were indicators of a technology requiring coverage.

**Figure 2.**
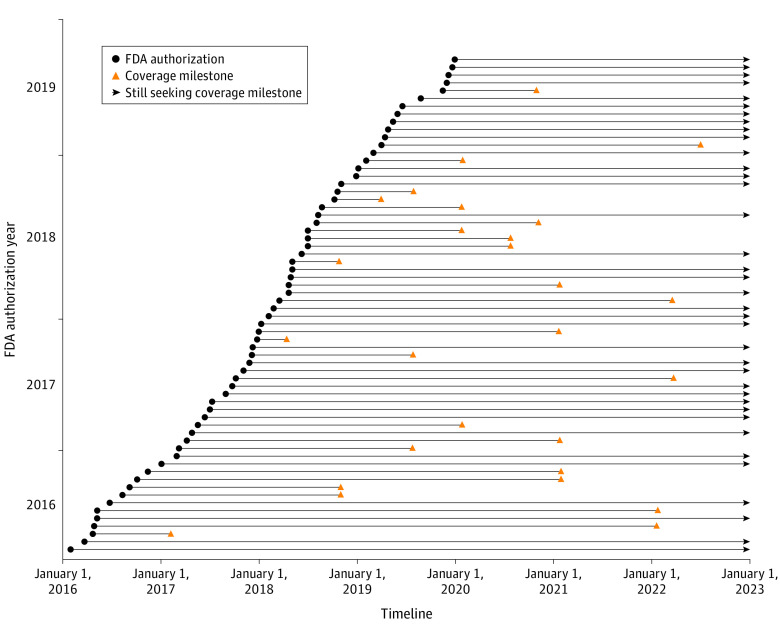
Time Spent Seeking a Coverage Milestone Is Variable FDA indicates US Food and Drug Administration. Among 64 analyzed technologies that required establishment of new Medicare coverage, the time spent seeking a coverage milestone varied from less than 91 days to about 7 years. The data represented by the arrowheads are right-censored because a coverage milestone was not achieved within the follow-up period.

**Figure 3.**
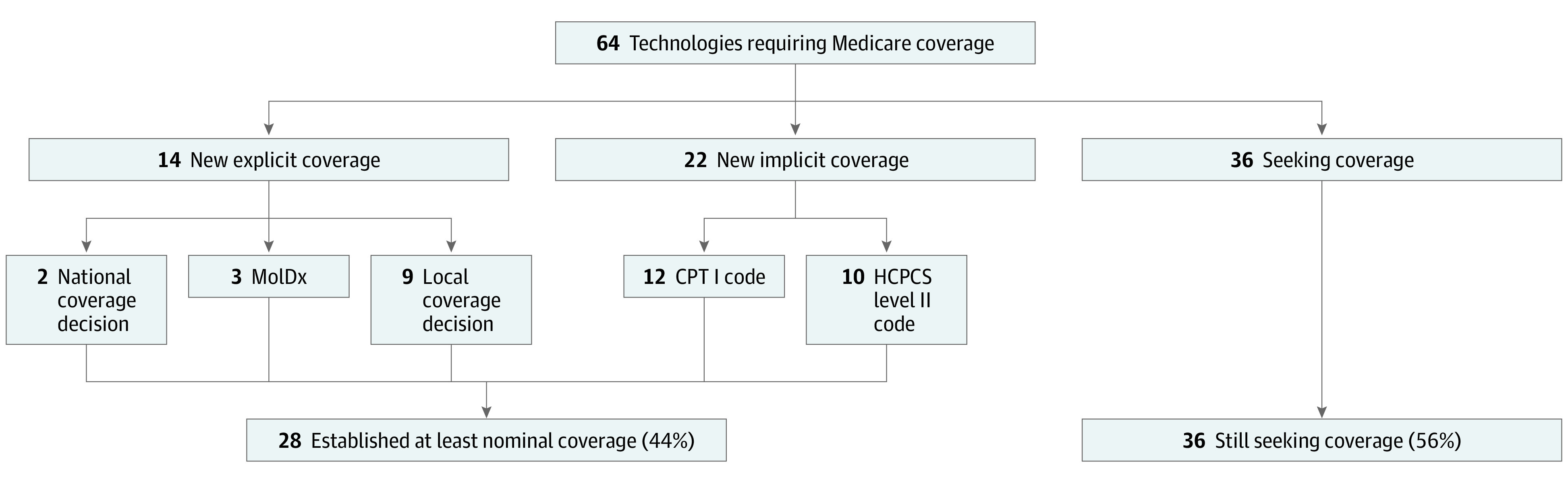
Establishing Implicit Coverage Is a More Frequent Path to a Coverage Milestone CPT 1 Indicates common procedural terminology; HCPCS, Healthcare Common Procedure Coding System; MolDx, molecular diagnostic services; Among 281 technologies in the analysis set were US Food and Drug Administration authorized during the enrollment period through the premarket approval and de novo pathways. A small subset of these technologies (n = 64) did not have appropriate billing codes or coverage available, thus new coverage was sought. Explicit coverage through new coverage determinations covered reimbursement of 14 technologies; implicit coverage through new codes supported reimbursement for 22 technologies. Of the 28 technologies that reached at least nominal coverage, 8 (29%) achieved both implicit and explicit coverage milestones.

For the 64 novel technologies, timelines from FDA authorization to at least nominal coverage are shown in (Figure 2). The shortest time to achieve a coverage milestone was 91 days and the longest within the limited study period was about 7 years (2546 days). Two technologies were evaluated through the Coverage with Evidence Development (CED) and CMS-FDA Parallel Review programs. Overall, 28 (44%) of the novel technologies in the cohort reached at least nominal coverage by the conclusion of the study period. Of those that achieved nominal coverage, 14 (50%) reached explicit coverage through an NCD, Molecular Diagnostic Services (MolDx) decision, or a minimum of LCDs; whereas 22 (79%) reached implicit coverage through assignment of a new billing code. Overall, 8 (29%) achieved both implicit and explicit coverage (Figure 3).

**Figure 4.**
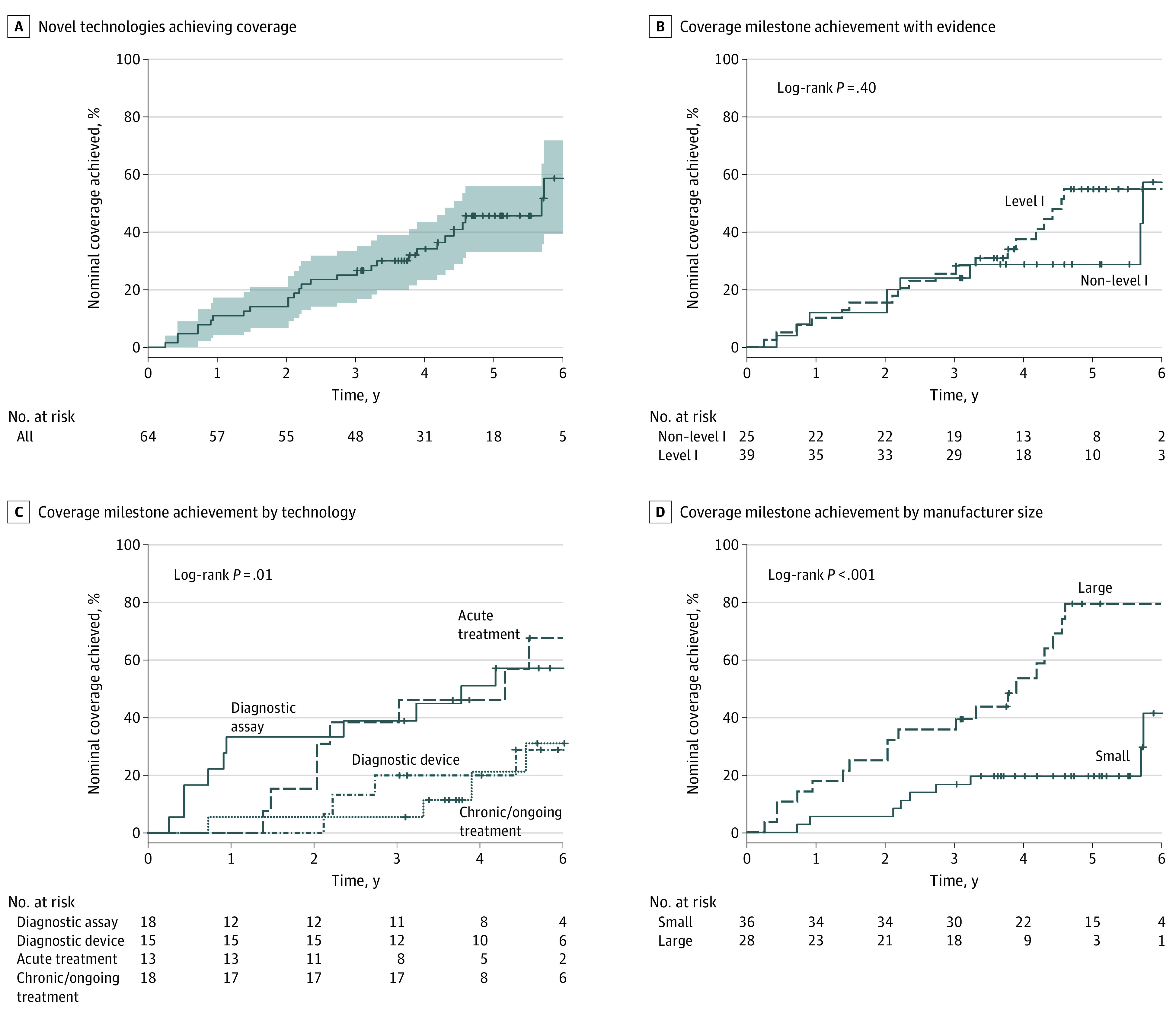
Factors Associated With Time to Achieve at Least Nominal Coverage A, Probability of achieving coverage among all technologies requiring new Medicare coverage. The median time to achieve new coverage was 5.7 years (2077 days). The shaded area represents the 90% CI. B, Analysis of coverage milestone achievement among technologies with level 1 (dashed lines) gold-standard clinical evidence and non–level 1 (solid lines) evidence at FDA authorization (log-rank *P* = .40). C, Analysis of coverage milestone achievement by technology type. Diagnostic assays (solid lines), acute treatments (dotted lines), diagnostic devices (dashed lines), and chronic/ongoing treatments (dot-dashed lines) (log-rank *P* = .01). D, Analysis of coverage milestone achievement by commercial manufacturer size. Small manufacturers (<200 employees; solid lines) and large manufacturers (≥200 employees; dashed lines) (log-rank *P* < .001). For all curves, tick marks “+” within curves represent right-censored dropouts of technologies at the cut-off of the study window (December 31, 2022).

Across the 64 novel technologies seeking new coverage, 18 (28%) reached a coverage milestone within 3 years of FDA authorization. The apparent coverage probability for a novel technology at 1, 3, and 5 years after FDA authorization was 10.9% (90% CI, 5.48%-19.9%), 25.0% (90% CI, 16.6%-35.6%), and 40.6% (90% CI, 30.4%-51.7%), respectively. The time at which 50% of the sample had achieved at least nominal coverage was 5.7 years (90% CI, 4.4-not applicable [NA] years) after FDA authorization, where the upper bound of the confidence interval could not be estimated given the limited number of novel technologies that achieved coverage during the study period (Figure 4A). Potential covariates for coverage milestone achievement were also investigated including strength of clinical evidence, type of technology, and size of commercial manufacturer. For clinical evidence, most technologies (n = 39, 61%) had level 1, gold-standard, clinical evidence at FDA authorization; the remaining technologies had lower levels of clinical evidence (eg, nonrandomized or single-arm trials). Evaluating the 3- and 5-year coverage milestone achievement probabilities, we observed similar 3-year coverage probabilities 25.6% (90% CI, 15.0%-39.8%) and 24.0% (90% CI, 11.5%-42.4%) for technologies with and without level 1 evidence, respectively. Five-year coverage probabilities trended toward greater difference with 48.7% (90% CI, 34.9%-62.7%) and 28.0% (90% CI, 14.4%-46.4%) for technologies with and without level 1 evidence. However, there was no statistically significant difference in time to coverage comparing technologies with and without level 1 evidence (log-rank, *P* = .40) (Figure 4B). For diagnostic assays, diagnostic devices, acute treatments, and chronic or ongoing treatments, we calculated the coverage probabilities at 3 and 5 years after FDA authorization. Three-year coverage probabilities were 38.9% (90% CI, 20.5%-60.6%), 20.0% (90% CI, 6.33%-44.4%), 38.5% (90% CI, 17.3%-64.2%), and 5.56% (90% CI, 0.38%-25.2%), respectively. At 3 years, diagnostic assays and acute treatments trended toward greater coverage than diagnostic devices and chronic or ongoing treatments. This was further pronounced at 5 years where probabilities were 55.5% (90% CI, 34.3%-75.1%), 26.7% (90% CI, 10.4%-51.2%), 61.5% (90% CI, 35.8%-82.7%), and 22.2% (90% CI, 8.58%-44.3%), respectively. Time to coverage was statistically different across product types (log-rank, *P* = .01) (Figure 4C). Last, manufacturer size showed strong association with time to coverage (log-rank, *P* < .001) (Figure 4D). Post FDA authorization, coverage probabilities were 5.56% (90% CI, 1.19%-17.3%), 16.7% (90% CI, 7.89%-30.7%), and 19.4% (90% CI, 9.88%-33.8%) for small manufacturers and 17.9% (90% CI, 7.76%-34.4%), 35.7% (90% CI, 21.2%-53.0%), and 67.9% (90% CI, 50.5%-81.7%) for large manufacturers at 1, 3, and 5 years, respectively.

## Discussion

This study examines 281 technologies approved or cleared through the FDA’s PMA and de novo pathways, as well as 510(k) devices with breakthrough designation between January 1, 2016, and December 31, 2019. Of the 281 technologies included in the sample, 171 were technologies that required distinct reimbursement. Of these, 107 used existing reimbursement processes, whereas 64 technologies were novel and required establishment of a new reimbursement process. For this later subset, at least nominal Medicare coverage supportive of beneficiary availability was achieved by only 28 (44%), with a median of 5.7 years (90% CI, 4.4-NA years). Just 6 (9%) novel technologies had achieved a coverage milestone within 2 years, and 18 (28%) within 3 years.

This study also found considerable variability in time to coverage milestone achievement. Among 3 hypothesized factors for such variability, manufacturer size showed the most striking difference and suggests a disproportionate burden for small manufacturers. This could be for several reasons, including the financial ability for larger manufacturers to hire individuals with expertise navigating the path to milestones or to design and undertake additional analysis suitable for health technology assessment. Another striking difference was that diagnostic assays reached at least nominal coverage more rapidly than other types of devices. This could be reflective of the effects of the MolDx Program. The MolDx Program was established to make coverage determinations specifically for molecular diagnostic tests and establishes coverage for 6 MAC jurisdictions at one time. Finally, there was no association between coverage milestone achievement and level of evidence developed for FDA authorization. This finding suggests that it is not necessarily the clinical evidence that is responsible for the lengthy time to coverage and perhaps points to other factors such as the coverage determination process itself, limited resources at CMS to support timely review, or some aspect within the level 1 studies that did not satisfy CMS’ reasonable and necessary standard. Certainly, as supported by other investigators, the lack of clear and predictable evidence criteria required by CMS for coverage determinations is a known issue for new devices and diagnostics.^24,25^

Over the past few decades, government agencies including the FDA and CMS have developed acceleration programs designed to close the gap between FDA authorization and new Medicare coverage. Programs like CED, the FDA Payer Communication Task Force, and Parallel Review have been implemented to facilitate greater access to emerging technologies. However, recent reviews suggest that these current programs fall short of their intended goals, with effects limited by low utilization and lack of clarity on CED program completion.^26,27^ We note that the Parallel Review and CED programs were only used in 2 of the 64 novel technologies examined in this cohort, corroborating review findings.

A recent CMS rule proposal for Medicare Coverage of Innovative Technologies (MCIT) offered an approach in which selected technologies would be guaranteed temporary coverage for up to 4 years after FDA authorization.^28^ Although the MCIT pathway was repealed, it inspired subsequent conversations and bipartisan legislative proposals, including the Transitional Coverage of Emerging Technologies (TCET) program. Many stakeholders have supported a new pathway that will support accelerated Medicare coverage for novel medical technologies linked to specific clinical evidence collection for Medicare beneficiaries. Such programs would provide support for clinicians to determine benefits, risks, and efficacy in the complex Medicare population. The results of this study suggest there is a need for a dedicated pathway that closes the substantial coverage gap demonstrated herein and provides a process for early communication between CMS and manufacturers that informs evidence development resulting in final coverage determinations.

### Strengths and Limitations

Among the strengths of the current analysis is its reliance on a timely and comprehensive set of data including a cross-section of technologies receiving FDA authorization in recent years and a range of different reimbursement pathways.

At the same time, this study is subject to several limitations. First, the analysis and interpretation of data largely adopted the perspective that access to technologies authorized by FDA—and therefore deemed safe and effective—is a desirable objective for both patients and society. This perspective is supported by ongoing initiatives intended to create an accelerated approval pathway.^28^ Nevertheless, additional postauthorization clinical evidence may be necessary for the Medicare population, and it is appreciated that such evidence collection takes time that might be well justified. Second, despite its size, the studied sample did not include the full scope of FDA 510(k) technologies, which represent the bulk of technologies authorized by the FDA each year. As such, the findings apply primarily to true novel technologies as opposed to technologies that are deemed substantially equivalent to technologies already authorized. Third, although industry experts provided information for the definition of coverage milestones associated with at least nominal coverage, their direct involvement with the studied technologies could have introduced potential bias. Fourth, calculation of time to coverage was based on a Kaplan-Meier survival analysis as opposed to direct measurement of time-to-coverage milestones for each technology. However, this is a well-established approach to account for right-censored data, while capturing the full analysis cohort. Finally, technologies that require a new Medicare benefit category, such as digital therapeutics, were excluded from analysis as were technologies that used temporary supplemental payment programs administered through C-codes, such as the Transitional Pass-Through payment and New Technology Add-on Payment.

## Conclusions

In this cross-sectional study, 64 medical devices and diagnostics among 281 technologies authorized by the FDA from 2016 to 2019 required new Medicare coverage. The median time to at least nominal coverage was 5.7 years (90% CI 4.4-NA years). The time required to establish at least nominal coverage results in uneven beneficiary availability and stretches longer than the time to average FDA authorization.^2^ These data highlight the need for establishment of a more efficient and timely reimbursement process for novel FDA-authorized medical devices and diagnostics.

## Notes

Supplement 1. eMethods. Determination of at Least Nominal Medicare Coverage eTable 1. Distribution of Technologies by FDA Reviewing Committee eTable 2. Type of Technology in the Total and Analysis Cohorts

Supplement 2. Data Sharing Statement
