# Supplementary material for: Time From Authorization by the US Food and Drug Administration to Medicare Coverage for Novel Technologies
Source: JAMA Health Forum. 2023 Aug 4;4(8):e232260. doi: 10.1001/jamahealthforum.2023.2260 (PMC10403784; doi:10.1001/jamahealthforum.2023.2260)
Supplement: Supplement 1. — eMethods. Determination of at Least Nominal Medicare Coverage eTable 1. Distribution of Technologies by FDA Reviewing Committee eTable 2. Type of Technology in the Total and Analysis Cohorts [file jamahealthforum-e232260-s001.pdf]

Supplemental Online Content

Sexton ZA, Perl JR, Saul HR, et al. Time from authorization by the US Food and Drug Administration to Medicare coverage for novel technologies. *JAMA Health Forum*. 2023;4(8). doi:10.1001/jamahealthforum.2023.2260

eMethods. Determination of at Least Nominal Medicare Coverage

eTable 1. Distribution of Technologies by FDA Reviewing Committee

eTable 2. Type of Technology in the Total and Analysis Cohorts

This supplemental material has been provided by the authors to give readers additional information about their work.

© 2023 Sexton ZA et al. *JAMA Health Forum*.

eMethods. Determination of at Least Nominal Medicare Coverage

Industry experts from 25 companies and familiar with 40 technologies in the total cohort were interviewed to contextualize the current technology reimbursement in the form of a narrative sequence of events. Respondents were asked to relate only publicly available information. First, they were asked to relate the history of the coding, coverage and payment milestones that occurred following FDA authorization, then to provide the sequence of evidence collected and published following FDA authorization. All milestones and publications were verified to ensure accurate recall. 21 technologies fell within established pathways to reimbursement such that a CPT I code or HCPCS Level II code description sufficiently described a procedure or diagnosis where the technology could be utilized (implicit coverage) and in some cases a Medicare coverage decision defined the conditions for use of the technology (explicit coverage). 17 technologies did not have established coverage, and two technologies were no longer commercially available.

The authors utilized the information collected in the interviews along with their own experience with coding, coverage and payment to establish the threshold for at least nominal Medicare coverage explained in Box 1. ICD-10-PCS were excluded from analysis because they are used to describe in-patient procedures with no product-specific coverage or payment. Products receiving temporary supplemental payment through the New Technology Add-on Payment (NTAP) program or Transitional Pass-Through (TPT) payment are tracked using HCPCS Level II C-codes, and HCPCS Level II K-codes are used to assign temporary payment rates for DME products. These, too, were excluded from the definition of at least nominal coverage. It was the opinion of the authors that the temporary nature of these programs and codes did not establish at least nominal coverage supportive of physician utilization and beneficiary availability.

© 2023 Sexton ZA et al. *JAMA Health Forum.*

eTable 1. Distribution of Technologies by FDA Reviewing Committee

Reviewing Committee, number (%)	Total cohort (n=281)	Analysis Cohort (n=64)
Cardiovascular	66 (23)	9 (14)
Microbiology	33 (12)	10 (16)
Neurology	29 (10)	13 (20)
General and Plastic Surgery	20 (7.1)	4 (6.3)
Pathology	21 (7.5)	3 (4.7)
Clinical Chemistry	18 (6.4)	4 (6.3)
Gastroenterology / Urology	18 (6.4)	5 (7.8)
Ophthalmic	15 (5.3)	7 (11)
Orthopedic	11 (3.9)	1 (1.6)
Anesthesiology	8 (2.8)	1 (1.6)
Immunology	7 (2.5)	1 (1.6)
Obstetrics & Gynecology	6 (2.1)	0
Radiology	6 (2.1)	0
Toxicology	5 (1.8)	3 (4.2)
None listed (Division of Immunology and Hematology Devices)	5 (1.8)	0
Ear, Nose and Throat	4 (1.4)	2 (3.1)
Hematology	4 (1.4)	1 (1.6)
Molecular Genetics	2 (0.7)	0
General Hospital	2 (0.7)	0
Dental	1 (0.4)	0

eTable 2. Type of Technology in the Total and Analysis Cohorts

Type of Technology, number (%)	Total cohort (n=281)	Analysis Cohort (n=64)
Acute Treatment	86 (31)	13 (20)
Chronic/On-going Treatment	73 (26)	18 (28)
Diagnostic Assay	75 (27)	18 (28)
Diagnostic Device	47 (17)	15 (24)

© 2023 Sexton ZA et al. *JAMA Health Forum.*
