# Supplementary material for: Time From Authorization by the US Food and Drug Administration to Medicare Coverage for Novel Technologies
Source: JAMA Health Forum. 2023 Aug 4;4(8):e232260. doi: 10.1001/jamahealthforum.2023.2260 (PMC10403784; doi:10.1001/jamahealthforum.2023.2260)
Supplement: Supplement 2. — Data Sharing Statement [file jamahealthforum-e232260-s002.pdf]

Data Sharing Statement

Sexton. Time From Authorization by the US Food and Drug Administration to Medicare Coverage for Novel Technologies. *JAMA Health Forum*. Published August 04, 2023. doi:10.1001/jamahealthforum.2023.2260

Data
Data available: Yes
Data types: Data (not involving human participants)
How to access data: Available by email to corresponding author
When available: With publication

Supporting Documents
Document types: Statistical/analytic code
How to access documents: Available by email to corresponding author
When available: With publication

Additional Information
Who can access the data: anyone requesting the data
Types of analyses: any purpose
Mechanisms of data availability: with investigator support
Any additional restrictions: NA
